# P-1662. Post-Acute Sequelae of COVID-19 and Post-COVID-19 Vaccination Syndrome: A Comparison of Clinical Immune Biomarkers

**DOI:** 10.1093/ofid/ofaf695.1837

**Published:** 2026-01-11

**Authors:** Thomas Heisler, Lawrence Purpura, Michael Yin, Steven Palmer, Jayesh Shah, Ga Young Seo, Abigail Graham, Antonia Sturiza Saint Jean, Xiomara Javier, Giselle S Pinto, Joan Bosco, Karl Reis, Magdalena E Sobieszczyk, Amanda R Castillo

**Affiliations:** UC Davis Internal Medicine Residency, New York, NY; Columbia University Medical Center, Brooklyn, NY; Columbia University Medical Center, Brooklyn, NY; Columbia University Medical Center, Brooklyn, NY; Columbia University Medical Center, Brooklyn, NY; Columbia University College of Dental Medicine, New York, New York; Columbia University Vagelos College of Physicians and Surgeons, New York, New York; Columbia University Medical Center, Brooklyn, NY; Columbia University Irving Medical Center, New York, New York; Columbia University Irving Medical Center, New York, New York; Columbia University Irving Medical Center, New York, New York; University of Washington, Seattle, Washington; Division of Infectious Diseases, Department of Medicine, Vagelos College of Physicians and Surgeons, New York-Presbyterian Columbia University Irving Medical Center, New York, NY, USA, New York, New York; Columbia University Medical Center, Brooklyn, NY

## Abstract

**Background:**

Post-acute sequelae of COVID-19 (PASC), and post-COVID-19 vaccination syndrome (PVS) present overlapping but distinct clinical challenges. Immunologic biomarker abnormalities have previously been established in PASC but less so in PVS. This study aims to distinguish immunologic biomarker patterns in PASC and PVS.

**Figure 1:**
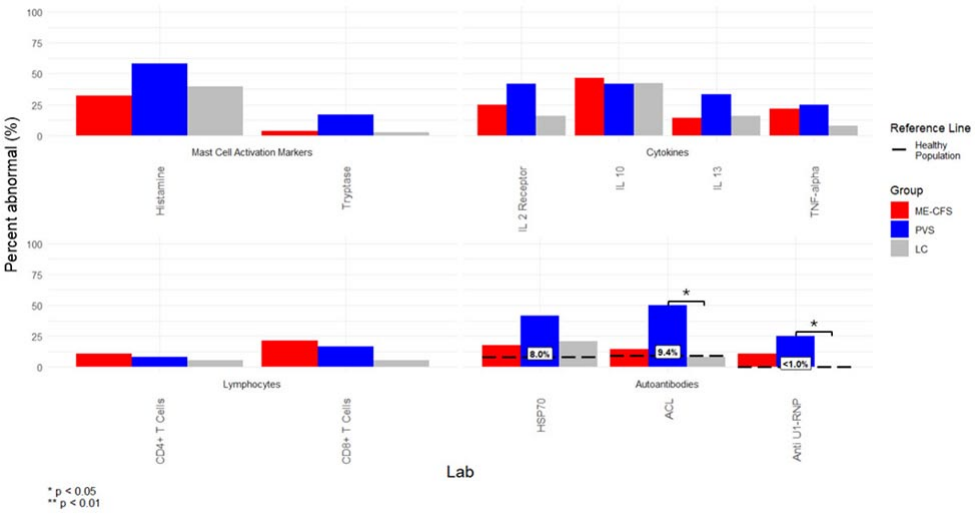
Clinical lab abnormality level, as defined by percent of patients with abnormal lab result at anytime (initial visit or follow-up), compared between the long covid positive for MECFS (MECFS), post-vaccination syndrome (PVS), and long covid negative for MECFS (LC) groups and previously reported positivity rates in healthy populations. Histamine: plasma histamine level, Tryptase: plasma tryptase level, IL 2 receptor: interleukin 2 receptor (CD25), IL 10: interleukin 10, IL 13: interleukin 13, TNF-alpha: tumor necrosis factor alpha, CD4+ T Cells: quantitative CD3+/CD4 lymphocytes, CD8+ T Cells: quantitative CD8+ lymphocytes, HSP70: heat shock protein-70 IgG western blot, ACL: quantitative anticardiolipin IgM, Anti U1-RNP: quantitative ribonucleic protein extranuclear antibody IgG. Reference ranges: Histamine: 0-8 mmol/L, Tryptase: <=10.9 ug/L, IL 2 receptor 175.3-858.2 pg/mL, IL 10: <=2.8 pg/mL, IL 13 <=2.3 pg/mL. TNF-alpha <=7.2 pg/mL, CD4+ T Cells: 393-1489 cells/uL, CD8+ T Cells: 148-788 cells/uL, HSP70: Negative, ACL: <=12 MPL, Anti U1-RNP: <1.0 U

**Methods:**

This cross-sectional study analyzed 78 participants from a Long COVID clinic divided into PASC with myalgic encephalomyelitis/chronic fatigue syndrome (MECFS, n = 28), PASC without MECFS (LC, n = 38), and PVS (n = 12) groups. The proportion of laboratory abnormality for each group was defined as the number of participants with a result greater than laboratory reference range at any point in their clinic course, over the total number of participants in the group. Statistical significance was determined using logistic regression controlling for age, sex, race, ethnicity, and history of autoimmunity.

**Results:**

The groups did not differ significantly in all demographic factors aside from median age (MECFS 38, PVS 58, LC 48; p = 0.04). All groups showed a high proportion of anti-HSP-70 antibody positivity (MECFS 17.9%, PVS 41.7%, LC 21.1%) greater than previously reported in healthy populations (8%). The PVS group showed a significantly higher proportion of anti-cardiolipin IgM (PVS 50.0%, LC 7.9%; p = 0.04) and anti U1-RNP (PVS 25.0%, LC 0.0%; p = 0.004) autoantibody positivity compared to the LC group. In all groups, elevations in IL 2 receptor, IL 10, IL 13, and TNF-α were common (Figure 1). The proportion of absolute CD8+ T-cell above normal ( > 788 cells/µL) appeared to be higher in the MECFS group (MECFS 21.4%, PVS 16.7%, LC 5.3%), although not significant (Figure 1).

**Conclusion:**

The high level of autoantibody positivity in the PVS group suggests a potential role of autoimmunity. Few studies have reported the presence of HSP-70 autoantibody in PASC or PVS, yet positivity was high in all groups, warranting further investigation into its potential role in pathogenesis. PVS and PASC show overlapping patterns of cytokine and T cell levels; however, future studies should investigate functional T cell markers as a measure of immune activation. Overall, this study is limited by small sample size and lack of control group. Future studies are needed to validate these results.

**Disclosures:**

Lawrence Purpura, III, MD, MPH&TM, Regeneron: Grant/Research Support

